# Communication in cancer care: psycho-social, interactional, and cultural issues. A general overview and the example of India

**DOI:** 10.3389/fpsyg.2014.01332

**Published:** 2014-11-17

**Authors:** Santosh K. Chaturvedi, Fay J. Strohschein, Gayatri Saraf, Carmen G. Loiselle

**Affiliations:** ^1^Department of Psychiatry, National Institute of Mental Health and NeurosciencesBangalore, India; ^2^Ingram School of Nursing, McGill UniversityMontreal, QC, Canada; ^3^Segal Cancer Center, Jewish General HospitalMontreal, QC, Canada; ^4^Psychiatry, National Institute of Mental Health and NeurosciencesBangalore, India

**Keywords:** communication, cancer, psycho-oncology, culture

## Abstract

Communication is a core aspect of psycho-oncology care. This article examines key psychosocial, cultural, and technological factors that affect this communication. Drawing from advances in clinical work and accumulating bodies of empirical evidence, the authors identify determining factors for high quality, efficient, and sensitive communication and support for those affected by cancer. Cancer care in India is highlighted as a salient example. Cultural factors affecting cancer communication in India include beliefs about health and illness, societal values, integration of spiritual care, family roles, and expectations concerning disclosure of cancer information, and rituals around death and dying. The rapidly emerging area of e-health significantly impacts cancer communication and support globally. In view of current globalization, understanding these multidimensional psychosocial, and cultural factors that shape communication are essential for providing comprehensive, appropriate, and sensitive cancer care.

## INTRODUCTION

Communication is an important aspect of psycho-oncology. Optimal communication in cancer care requires that the actors involved have adequate levels of knowledge, adequate communication skills, and an open attitude (Surbone, 2008) to facilitate the flow of information, emotions, and supportive interpersonal behavior. In addition, because cancer is often associated with suffering, aggressive treatment, and bodily harm and stigma, the content of communication can be complex. This paper offers key insights into important phenomena that affect the quality of communication in cancer care. These include psychosocial, demographic and cultural factors, highlighting cultural aspects of cancer care in India as a salient example, and technological advances in communication and supportive cancer care. Each is reviewed in turn.

## PSYCHOLOGICAL, SOCIO DEMOGRAPHIC AND CULTURAL FACTORS IN HEALTH CARE

Socio-demographic characteristics of both patients and health care professionals can have profound influences on communication in cancer care. Age, gender, level of education, socio-economic level, and ethnicity are five such characteristics that have been found to influence patterns of communication. For instance, age, gender, and education have been shown to be significantly associated with patient preferences for communication of prognostic information, with younger, female, and more highly educated patients preferring more detailed information and emotional support (Fujimori and Uchitomi, 2009).

Disparities in cancer care and health outcomes among patients with varying socio-demographic characteristics also can be traced to poor communication (Siminoff et al., 2006). In a study of newly diagnosed breast cancer patients, disparities in communication patterns were found according to age, education, income, and race: patients who were older, with less education, less aﬄuent, and members of racial or ethnic minorities are found to be provided with less information, and also expressed less about their own illness experience to their physician, resulting in poorer communication that may impact treatment decision making (Siminoff et al., 2006). Disparities in patterns of communication based on patient socio-demographic characteristics may be the result of ignorance of social or cultural norms, patient behaviors and attitudes that may be correlated with certain psychosocial characteristics, or assumptions and stereotypes (Roter and Hall, 2006). In addition to its impact on patient satisfaction, adherence, psychological adjustment, quality of life, and treatment decision making (Arora, 2003), poor communication, such as that relating to socioeconomic disparities, can result in considerable economic costs for the health care system (Thorne et al., 2005).

Older adults with cancer are at increased risk of experiencing poor communication with health care professionals. Based on differing historical contexts, older adults may have different attitudes and beliefs about cancer than younger adults, as well as less health literacy and more cognitive or sensory deficits, thus introducing multiple communication challenges (Greene and Adelman, 2003). Characteristics of the health care system itself also create communication challenges for older adults, such as visits being too short to discuss the complex concerns of those with multiple chronic conditions (Greene and Adelman, 2003). Older patients are less likely to request or receive information, and are often perceived by health care professionals as wanting less information (Hagerty et al., 2005). Studies have shown that older adults want less detailed information than younger adults, but may want specific information about life expectancy (Fujimori and Uchitomi, 2009) and potential impact of treatment on daily functioning (Puts et al., 2012). Vast variation among older adults, however, requires clear assessment of information desires and needs to develop an individualized approach to information provision (Posma et al., 2009), avoiding ageist attitudes and stereotypes that may themselves impair communication.

Culturally dominant masculinities, which are reflected in behaviors that evidence strength, independence, and invulnerability, increase risk behavior and are contrary to health-promoting behavior; femininities, such as asking for help and caring for one’s body, are consistent with health-promoting behaviors (Courtenay, 2000). In addition, women demonstrate a more relation-oriented approach to communication, as compared to the more task-oriented approach of men (Brink-Muinen et al., 2002). This is evidenced by female and male physicians differing in their communication patterns with patients, with female physicians showing more affective behavior, partnership statements, information provision, treatment options, and attention to psychosocial concerns, and male physicians providing more instructions, warnings and suggestions (Brink-Muinen et al., 2002). It is also evidenced by patients, with female patients expressing more emotions, providing longer responses to questions, and preferring emotionally supportive communication, and male patients providing more facts, getting more attention, and tending to be better liked by their physicians (Brink-Muinen et al., 2002). Women generally have been found to want and receive more information and emotional support in communication interactions (Hagerty et al., 2005; Roter and Hall, 2006; Fujimori and Uchitomi, 2009). These gender differences in communication are thought to contribute to differences in understanding about cancer, with women being more likely than men to acknowledge that their illness was incurable, having a better understanding of the advanced nature of their cancer, and more frequently reporting having discussions about life expectancy with their oncologist (Fletcher et al., 2013). Gender, however, does not only reside in the person, but also in social interactions and behaviors that are defined as gendered (Lyons, 2009). There may be power differences between opposite-gender patient-physician dyads that affect communication patterns (Brink-Muinen et al., 2002), particularly in cultures where traditional gender roles are more dominant.

## COMMUNICATION IN CANCER CARE – CULTURAL FACTORS

Cultures vary across countries in terms of economic status, education and resources, as well as by traditional and family values, and religious or spiritual aspects pertaining to illness and health. Health care providers must be attentive and responsive to cultural factors that shape worldview, values, and ethos (Engebretson et al., 2001). In this way, culture shapes beliefs about health, illness, death, and dying; expectations concerning disclosure of diagnosis and prognosis; family decision making roles; language; and perspectives concerning complementary and alternative medicine. These all have a direct impact on communication in cancer care.

Provider/patient/family communication in different sociological and institutional environments is shaped by the varied multi-cultural societies within and across these environments. Individuals with cancer often experience significant emotional distress as a result of their diagnosis. They are also asked to process complex information related to their specific diagnosis and treatment options, and to be involved in cancer-related decision making. All stakeholders including providers, patients, and family members are called upon to consider cancer-related issues, with providers and family members striving to support the affected individual (Engebretson et al., 2001). When differing cultural backgrounds of those involved are added to the cancer care situation, effective communication can be challenged. Though the cultural variations may be noted globally, those of one country, India, have been taken as an illustration for such variations.

## CULTURAL FACTORS IN CANCER CARE IN A TRADITIONAL COUNTRY, (INDIA)

### BELIEFS RELATED TO HEALTH AND ILLNESS

Beliefs vary across countries and regions. These beliefs are, in large part, shaped by religious traditions, which may take on “some of the essential elements of culture” (Martsolf, 1997, p. 232). It is often difficult to distinguish between beliefs stemming from religious affiliation from those associated with ethic or cultural background (Miller, 1995). Common religions endorse different views related to health, disease, illness, death, and overall health care. In Hinduism, cancer is considered by many to be due to *past sins or karma.* In addition to religious beliefs, cultural views can shape understanding of the origins of illness, particularly cancer. In many cultures, traditional societal, religious, and dietary factors are understood as direct causes of cancer, rather than the more genetic or biological factors. In Western cultures, individuals are expected to gain mastery over nature, and be involved in an active struggle to “fight cancer” and regain health. In Eastern cultures, individuals are expected to live in harmony with nature, with passivity and fatalistic views being common. In many traditional societies, disease is considered God’s punishment, or one’s Karma (Chaturvedi and Chandra, 1998). Patients and providers must work through potentially contradictory views and beliefs related to cancer to achieve common therapeutic goals (Surbone, 2008).

### RELIGIOUS AND SPIRITUAL CARE

Spirituality differs from religion. It may be defined in terms of holding a sense of meaning and purpose in life, values, connection with self, others, and a higher power, and sense of becoming (Martsolf, 1997). It can play an important role in the cancer experience, with spiritual care being increasingly seen as an essential component of cancer care. It plays a particularly prominent role in the developing world where medical and comfort resources remain limited. In a study in an East Indian setting, spiritual dimensions were reported to be most important aspects of quality of life, in contrast to functional aspects (Chaturvedi, 1991). In a study that aimed to understand patients’ preferences regarding the role of doctor in facilitating a sense of peace, Best et al. (2014) found that doctors could facilitate a sense of peace and spiritual well-being by giving clear and honest information regarding what patients should expect. Religion, which may be seen as “a way of thinking about spiritual issues that is prescribed by a particular group of people” (Martsolf, 1997, p. 232), may shape approaches to spiritual care. Professionals working in palliative care settings in India employ diverse methods to improve quality of life of their patients by suggesting prayer, devotion, yoga, meditation, or philosophical pursuits, whereas the patients themselves seek abode in holy or religious places, akin to hospice towns or cities, to obtain mental and spiritual solace awaiting death (Chaturvedi, 2007).

### FAMILY ROLES

Increasingly it is recognized that “cancer is not only an individual quest, it is a family and support network experience” (Ballard-Reisch and Letner, 2003, p. 61). The degree and type of family involvement, however, varies across cultures (Surbone, 2008). In addition, health care providers’ expectations regarding the participation of patient and family members in cancer decision making also differs among cultures (Ballard-Reisch and Letner, 2003; Surbone, 2008). Broadly speaking, in traditional societies, the family is much more implicated in the cancer experience, with it being viewed as family’s disease, whereas in the West, it is viewed primarily in terms of the individual. In a traditional society such as India, the family plays a significant role in each stage of diagnosis and management of a chronic illness such as cancer. Family issues are related to support, conflicts, involvement in medical care, and sharing of financial burden. Family support is central to an individual’s experience with cancer, with strong family ties lessening personal responsibility and providing significant instrumental and emotional help in certain circumstances. A serious illness such as cancer may also unravel into family conflicts. Family involvement in medical care can be used in a positive manner for the benefit of the patient. In western cultures, the emphasis on autonomy, empowerment and individual responsibility may become a burden and adversely affect the patient (Chaturvedi, 1994; Ho, 2008).

### COLLUSION

In terms of communication, family dynamics can be responsible for collusion and selective disclosure of cancer information. Collusion is a common phenomenon in Indian settings (Chaturvedi et al., 2009), relatives often wish to know the truth about a potential cancer diagnosis but prefer that it be not disclosed to their ill family member seemingly to guard against patients’ fears, apprehension, and curiosity which, in turn, could enhance cooperation in adherence to treatment modalities. Paradoxically, most patients prefer to be told of their diagnosis. In a study by Muckaden et al. (2005), two thirds of women with cervical cancer had their diagnosis concealed by their husband or family members. The family elders often assumed that the women would be unable to cope with bad news or having to make informed decisions. Interestingly, toward end of life, collusion still persisted only in about 15% of these women. This tendency toward collusion does not persist across all cultural groups in India. In study Gautam and Nijhawan (1987), some subjects felt that sooner or later patients would find out about their disease, so facts should not be hidden from them.

The decision making by the key family decision maker is generally supportive, but at times may be perceived as an interference by the health care professionals and the patient themselves. This interference may adversely influence the treatment and outcome of the disease. Although collusion may impair the communication between the health professionals and family, its primary purpose is to protect the patient. On the negative side, collusion may be viewed as coming in the way of an individual’s autonomy, and may deprive him of the benefits of health services and care. On the positive side, collusion may defend and protect the patient from distress, and may minimize their concerns about the future. Relatives want to protect their loved ones suffering from cancer. Whenever collusion interferes with the care, it needs to be broken or handled.

Cross-culturally, a study by Holland et al. (1987), documented that 40% of oncologists revealed the cancer diagnosis to patients in Africa, Japan, France, Spain, and Italy, whereas 80% revealed it in Austria, Denmark, and other European countries. In the US, in keeping with patient’s right movement, all patients are informed. In a study done in Taiwan that looked at nurses’ truth- telling experiences, 70% responded that they had told the truth to patients about their diagnosis. In the remainder, the reasons cited for not doing so were collusion, considering it to not be part of one’s duty, and perceiving it as difficult (Huang et al., 2014). Shubha (2007) has discussed this aspect in the Indian and Chinese contexts and asserts that Western individualistic cultures tend to prioritize autonomy and self-determination in end-of-life care, which are reflected in the practices of advance care planning, informed consent, individual decision-making, and candid communication of the patient’s condition. In contrast, non-Western cultures, such as Indian and Chinese ones, are largely influenced by principles of beneficence and non-maleficence, with a focus on promoting patients’ welfare and precluding harm to patients (Searight and Gafford, 2005). These values cause them to favor patients’ sustenance of hope. Families may want to protect patients by not discussing death and end-of-life decisions directly, whereby encouraging collusion. There is an increasing trend toward full disclosure to patients all over the world due to greater patient expectations and increased physician openness.

### LANGUAGE BARRIERS

Language barriers introduce challenges into communication across cultures. Communicating in a relatively unfamiliar language imposes an additional burden on both the patient and the provider. It is not uncommon among patients with poor knowledge of the language of care to express doubts about having explained their symptoms accurately to their physician. The patient may feel misunderstood as his/her fluency and expressions while describing symptoms, may be different from that of the health professional. It is also difficult for health professionals to make an adequate assessment of the patient’s health status when language acts as a barrier (Engebretson et al., 2001). Patients may misperceive linguistic differences as affective distancing by the health care provider and become more emotionally withdrawn. These barriers tend to block the patients desire to ask questions and talk freely.

Differences in language may be more subtle than those pertaining to having a different mother tongue. Merriam-Webster’s dictionary defines language as “the words, their pronunciation, and the methods of combining them used and understood by a community.” A person may use different words or ways of expressing themselves that are not understood in the same way by the other individual. For example, vegetarianism or lay beliefs about diet, such as those concerning hot and cold foods in Ayurvedic belief, may have a dramatic impact on the way an individual understands and communicates about food and nutrition in relation to health. In addition, within the same language, words can vary in terms of their meaning and connotation: for example, “in certain cultures, ‘cancer’ is a ‘bad word’…or words such as ‘cancer’ or ‘depression’ do not even exist” (Surbone, 2008, p. 238). Therefore, the choice of words is important in cross-cultural communication.

Cultural and social factors may also shape communication styles and skills. For instance, whereas Western societies are often construed as psychologically “sophisticated” and valuing individuals’ propensity for introspection, other cultures, such as those in India, have been considered as favoring consensual/communal experience over intra-individual mindedness. Approaches to agreement and disagreement may also vary between cultures and between individuals (Ballard-Reisch and Letner, 2003). Thus, some of the cultural and social factors in health and disease vary based on communication styles and skills. For instance, the desire for communication or consultation varies between cultures and between individuals (Ballard-Reisch and Letner, 2003). In Western cultures, it is encouraged to talk to the doctors, while in other cultures, such as that in India, the doctor’s word is not questioned.

### NON-VERBAL BEHAVIOR

Non-verbal behavior is also central to effective communication. This again may vary across cultures and thus be misinterpreted by patient and/or provider, further contributing to communication barriers. Patients belonging to a particular cultural group may sense that the health team is acting with an unfamiliar set of norms, while the health team professionals might be unaware of the meaning of a patient’s peculiar behavior. Hence there is a need to negotiate amicably an acceptable approach to care, which can be particularly challenging. For example, in terms of cultural attitudes toward pain, people from one culture may have stoic self-control, those from another may wail and moan with the same pain stimulus, and those from a third culture may first say how terrible it is and then face it. A health care professional’s interpretation of these expressions of pain may influence the frequency and type of pain management offered. Authors studying patient perspectives in communication developed an interesting typology of errors in communication: “occasional misses” are errors within an overall appropriate communication and can be prevented by a discussion between patient and clinician and communication skills training; “systemic misunderstandings” refer to departures from appropriate pattern of communication, such as giving excessive information, or withholding information, excessive use of numbers and statistics to convey possibilities and likelihoods; and “repeat offenders” are clinicians whose communication patterns become a constant source of distress to the patients concerned (Thorne et al., 2013). Thus the provider’s willingness to attend to errors in communication can greatly impact the overall patterns of communication and the potential impact of these errors.

### COMMUNICATION ABOUT DEATH AND DYING

Talking about death and bereavement is also influenced by cultural background. Cultural differences arise in attitudes toward life and death, with many cultures holding bereavement rituals and other traditional activities around death and dying (Chaturvedi and Chandra, 1998). Health professionals in cancer care must acknowledge cultural and religious beliefs in relation to death and dying: for example, transmigration of soul in Hindus, the concepts of heaven or hell for Christians and Muslims, a high degree of involvement with death among Hispanics, and various mourning rituals in different communities (Mazanec and Tyler, 2003). Western cultures minimize death related rituals, with minimal expression of feelings. There may be conflict between the beliefs and rituals that are important to the patient and those that are practiced by the health care providers. For example, among Hindus, customs and rituals for a dying person include putting the person on the ground (and not the bed), pouring holy water (of Ganges or any other holy river) in the person’s mouth, and chanting hymns (Chaturvedi and Chandra, 1998). The health professionals need to be aware of the religious sentiments of the individual, family, and society to be able to appropriately address the patient’s concerns during end-of-life care. This may begin by creating opportunities for open communication about the beliefs and rituals that are important to the patient and family.

Although different religious groups tend to view, understand, and cope with end-of-life issues in cancer in various ways, there are also cross-cultural commonalities in perspectives, particularly in relation to the meaning of cancer in relation to death and dying. Different societies, place varying emphasis on different issues related to death and dying and this can have profound effects on their psychosocial care needs during this time. Thus, problems in end-of-life care may arise from cultural and/or religious differences between staff and cancer patients. The meaning and fears around various issues related to death and dying must also be explored and addressed by health care providers.

Patients in some cultures wish to die at home: on one hand, receiving caring at home may be perceived as meaning that the doctors have ‘given up,’ on the other hand, it may mean the patient is able to prepare for peaceful death in a familiar environment (Chandra et al., 1999). In some cultures, death at home is preferred, whereas in other cultures, death in hospitals is preferred. Do-not-resuscitate (DNR) directives are another context in which good end-of-life communication is crucial and are being increasingly used in certain countries. A study done in the US reported that the median time between signing a do-not-resuscitate order and death was 0 days for inpatients. Authors conclude that this may be a marker for delayed palliative care and suboptimal end-of-life communication between the patient and the doctor (Levin et al., 2008). Preferences of patients, relatives, or doctors may differ with respect to place of death and DNR directives. Ideally, the patient should be allowed to make an independent, informed choice, to exercise his preferences, on where, how and by whom he/she would like to be cared for, and where he/she would like to meet the end of life. In traditional joint family systems, this decision is often made by the relatives. Doctors in palliative care settings in India often face this difficulty, and are persuaded by the family to decide on the patient’s behalf (Sureshkumar and Rajagopal, 1996). Most physicians view communication of prognosis at the end-of-life as a process (Jackson et al., 2008; Roscoe et al., 2013).

## FUTURE ADVANCES IN COMMUNICATION IN CANCER CARE

Unprecedented and rapid development of various technologies to enhance communication and support in cancer care seems to have emerged. For instance, patients, and health care providers alike use the internet to gather and/or exchange information about health conditions, seek online guidance or support, and communicate with others with similar diagnoses. As such, information and communication technologies are poised to transform access to and participation in health and illness. The complexity of this challenge is being shaped by concomitant transformations and improvements in survivorship translating into long-term management of cancer and related care rather than sporadic treatment of acute conditions. This places greater emphasis on the role of home, family caregivers, and community as significant contributors to individuals’ cancer experience and wellbeing. The substrate of 21^st^ century healthcare will be computing and networking concepts, as well as technologies whose transformative potential is tempered by unresolved core challenges in designing and optimizing them in terms of their applicability for all involved. Various terms have recently been proposed to capture involvement of multiple stakeholders in e-health. These include e-patient, e-caregiver, e-group, e-nurse referring to health consumers who use technology (namely the internet) to gather information and support about a health condition of particular relevance to them. One can thus foresee the tremendous impact that these new trends will have on various modes of communication.

These technologies may be accessed nationally and internationally, bridging distances between population groups, nations, and countries. In a study that explored health and cancer information-seeking practices in the population of Puerto Rico, it was found that the internet was the most frequently used source of information and that females and the college educated were more likely to seek information (Tortolero-Luna et al., 2010). Therefore, ongoing development of online cancer technologies will require particular attention to the cultural, religious, and linguistic differences among the vast diversity of users. Future research will seek to document the nature of these changes as well as whether reliance on e-health solutions enhances, impedes or generally modifies the nature of cancer-related communication among patients, family members, and health care professionals.

Health care providers should strive toward increased awareness and knowledge of how culture affects the overall experience of illness and death in order to help create a mutually satisfactory care plan. Providing culturally competent care includes the use of proper communication skills to facilitate the exploration of patient and family perspectives and allows for mutual decision making (Longo and Slater, 2014). In future paradigms in patient-provider communication during cancer care, providers need to be aware of patient education levels, engage in behaviors that enhance trust, treat patients equally, respect religious beliefs, and reduce the difficulty level of the information (Song et al., 2014).

## CONCLUSION

Healthcare professionals in cancer care must strengthen their knowledge of factors that influence communication to improve their confidence and skills in providing optimal information and support to patients and their family members. This must include an awareness of the influence of socio-demographic and cultural factors that shape communication, as well as the rapid development of technologies that are transforming modes of communication. Socio-demographic factors including age, gender, socioeconomic status, education, and ethnicity can impact and increase the risk of poor communication. Poor communication, in turn, may explain disparities in health outcomes among groups with differing socio-demographic characteristics. Awareness of potential areas of communicative concern related to socio-demographic differences is necessary to provide effective and safe care.

The implications of understanding the multidimensional factors that affect communication between patients, family members, and providers are important in view of current globalization. It is crucial for health care providers to recognize the influence of socio-demographic, and cultural, factors to enhance culturally sensitive care for their increasingly diverse patients. By improving communication, knowledge about a person’s cultural background can also improve psychosocial care. Furthermore, it is equally important to attend to the ways in which the multidimensional factors affecting communication may also shape screening and measurement tools: for example, measures of quality of life must be culturally relevant (Stjernsward and Clark, 2004). Promoting cultural awareness and cultural competence among health care professionals, while at the same time encouraging recognition of the inter-individual differences (Mazanec and Tyler, 2003) and cultural factors affecting optimal communication, will improve their confidence and skills in providing comprehensive care for patients and families from different backgrounds.

Consideration of Indian cultural beliefs and practices provides a salient example of differences that may impact communication in cancer care. Exploration of differences related to beliefs about health and illness, religious, and spiritual care, family roles, disclosure of diagnosis and prognosis to the patient, verbal and non-verbal language, and death and dying highlights areas that may raise communication barriers that can have serious detrimental impact on a patients’ care and treatment, as well as on the family’s cancer care experience. Modes and substrates of cancer support are rapidly changing with advancement of information and communication technologies. Effective cultural sensitive communication relies on a variety of means to ensure information flow and satisfaction among health professionals, patients and families.

## Conflict of Interest Statement

The authors declare that the research was conducted in the absence of any commercial or financial relationships that could be construed as a potential conflict of interest.
